# The Evolution of Light Stress Proteins in Photosynthetic Organisms

**DOI:** 10.1002/cfg.221

**Published:** 2002-12

**Authors:** Mounia Heddad, Iwona Adamska

**Affiliations:** Department of Biochemistry and Biophysics Arrhenius Laboratories for Natural Sciences Stockholm University Stockholm S-10691 Sweden

## Abstract

The Elip (early light-inducible protein) family in pro- and eukaryotic photosynthetic organisms consists of more than 100 different stress proteins. These proteins accumulate in photosynthetic membranes in response to light stress and have photoprotective functions. At the amino acid level, members of the Elip family are closely related to light-harvesting chlorophyll *a/b*-binding (Cab) antenna proteins of photosystem I and II, present in higher plants and some algae. Based on their predicted secondary structure, members of the Elip family are divided into three groups: (a) one-helix Hlips (high light-induced proteins), also called Scps (small Cab-like proteins) or Ohps (one-helix proteins); (b) two-helix Seps (stress-enhanced proteins); and (c) three-helix Elips and related proteins. Despite having different physiological functions it is believed that eukaryotic three-helix Cab proteins evolved from the prokaryotic Hlips through a series of duplications and fusions. In this review we analyse the occurrence of Elip family members in various photosynthetic prokaryotic and eukaryotic organisms and discuss their evolutionary relationship with Cab proteins.


**Comparative and Functional Genomics**
*Comp Funct Genom* 2002; **3**: 504–510.
Published online in Wiley InterScience (www.interscience.wiley.com). **DOI:** 10.1002/cfg.221

**Conference Review**

### The evolution of light stress proteins in photosynthetic organisms

Mounia Heddad and Iwona Adamska*
*Department of Biochemistry and Biophysics, Arrhenius Laboratories for Natural Sciences, Stockholm University, S-10691 Stockholm, Sweden*

**Correspondence to:
Iwona Adamska, Department of Biochemistry and Biophysics, Arrhenius Laboratories for Natural Sciences, Stockholm University, S-10691 Stockholm, Sweden.
E-mail: Iwona@dbb.su.se*

Received: 7 September 2002
Accepted: 14 October 2002

### Abstract

**The Elip (early light-inducible protein) family in pro- and eukaryotic photosynthetic organisms consists of more than 100 different stress proteins. These proteins accumulate in photosynthetic membranes in response to light stress and have photoprotective functions. At the amino acid level, members of the Elip family are closely related to light-harvesting chlorophyll *a/b*-binding (Cab) antenna proteins of photosystem I and II, present in higher plants and some algae. Based on their predicted secondary structure, members of the Elip family are divided into three groups: (a) one-helix Hlips (high light-induced proteins), also called Scps (small Cab-like proteins) or Ohps (one-helix proteins); (b) two-helix Seps (stress-enhanced proteins); and (c) three-helix Elips and related proteins. Despite having different physiological functions it is believed that eukaryotic three-helix Cab proteins evolved from the prokaryotic Hlips through a series of duplications and fusions. In this review we analyse the occurrence of Elip family members in various photosynthetic prokaryotic and eukaryotic organisms and discuss their evolutionary relationship with Cab proteins. Copyright © 2002 John Wiley & Sons, Ltd.**

**Keywords:** **chloroplast; early light-induced proteins; evolution; high light-induced proteins; chlorophyll *a/b*-binding proteins; one-helix proteins; small Cab-like proteins; stress-enhanced proteins**

### Introduction

Plants and cyanobacteria respond to light stress by transient accumulation of light stress proteins from the Elip (early light-induced proteins) family [1]. These proteins are integrally located in photosynthetic membranes, spanning the membrane with either one (e.g. Ohps, one-helix proteins, called also Hlips; high light-induced proteins or Scps; small Cab-like proteins in prokaryotic organisms), two (e.g. Seps, stress-enhanced proteins) or three (e.g. Elips) $\alpha$-helices [1,6,10,15]. The members of the Elip family are closely related to chlorophyll *a/b*-binding (Cab) or fucoxanthin-chlorophyll *a/c*-binding proteins that form antenna systems around photosystems I (PS I) and II (PS II) in chlorophytes (green algae, mosses, ferns and higher plants) or chromophytes, respectively [4,19].

According to sequence conservation between Elip and Cab family members, all of these proteins are assumed to share a common evolutionary origin [7,19] and presumably have a similar structure. The three-dimensional structure of one Cab family member has been determined at 3.4 Å resolution by electron crystallography of two-dimensional crystals [12] showing that two of the three transmembrane $\alpha$-helices are held together by ion pairs formed by charged residues. Thus, it is expected that Elip family members will have a similar two-fold symmetry structure, since helices I and III of Elips and Cab proteins and the helix I of Seps and Ohps/Hlips/Scps are highly conserved in their amino acid composition. However, in order to form such structures, two-helix Seps and one-helix Ohps/Hlips/Scps need to form homo- or heterodimers.

In addition to similarities between Elip and Cab proteins there are also very pronounced differences. In contrast to Cab family members that

Copyright © 2002 John Wiley & Sons, Ltd.

are the most abundant proteins of the thylakoid membranes, Elips accumulate only transiently in substoichiometric amounts in response to physiological stress [1]. Moreover, a very unusual pigment composition and pigment-binding characteristics were reported for isolated Elips, such as a weak excitonic coupling between chlorophyll *a* molecules and an extremely high lutein content as compared with other chlorophyll-binding proteins [2]. Based on these features, a non-light-harvesting function has been proposed for Elip family members. It is believed that these proteins fulfil a photoprotective role within thylakoid membranes under light stress conditions, either by transient binding of free chlorophyll molecules and preventing the formation of free radicals, and/or by participating in energy dissipation [1,16].

In this review we analyse the evolutionary relationship of various members of Elip family in pro- and eukaryota and discuss the origin of eukaryotic Cab proteins.

### The distribution of Elip family members in photosynthetic organisms

To resolve the evolutionary relationship between one- two- and three-helix members from the Elip family and to investigate their relation with Cab proteins, we performed BLAST searches in various databases (**http://www.tigr.org**; **http://megasun.bch.umontreal.ca/ogmp/projects/other/cp_list.html**; **http://mips.gsf.de/proj/sputnik/oryza**; **http://www.jgi.doe.gov/JGI_microbial/html**) using the Elip consensus motif ERINGRLAMIGFVAALAVE, located in the first conserved transmembrane $\alpha$-helix [1,10], or full-length sequences of different Elip family members. Table 1 shows the distribution of the Elip family members across cyanobacteria, photosynthetic protists and plants, their sizes and their predicted secondary structures.

Searches in the databases of cyanobacteria revealed that multigene Elip families composed of eight Hlip/Scp members are present in the genome of *Nostoc* sp. PCC7120 or *Synechococcus* sp. PCC7942. *Prochlorococcus marinus* contained 10 predicted Hlip/Scp genes (Table 1). In contrast, only one member of Hlips/Scps was found in the Glaucocystophyta *Cyanophora paradoxa* or in red algae *Porphyra purpurea* and *Cyanidium caldarium*. The Cryptophyta *Guillardia theta* contained two Hlip/Scp members. All Hlips/Scps found in red algae or in Cryptophyta are encoded by the plastid genome (Table 1). In Glaucocystophyta the *Hlip/Scp* gene is located on a cyanelle genome (VL Stirewalt, CB Michalowski, W Löffelhardt, HJ Bohnert and D.A. Bryant, 1995; direct GenBank submission). Cyanelles are plastid-like organelles that resemble cyanobacteria in morphology, in the organization of their photosynthetic apparatus and in the presence of the peptidoglycan wall [14]. The recent acquisition of complete plastid genome sequences of Glaucocystophyta, Rhodophyta and Cryptophyta allowed us to search for Elip family members in these organisms. However, one should be aware that more Hlips/Scps may be discovered in the nuclear genomes of these algae.

To investigate whether the type of the photosynthetic antenna correlates with the number and/or type of Elip family members present in these organisms, we compared the antenna systems of red algae, Cryptophyta and the cyanobacteria *Nostoc* and *Synechococcus*. Both algal groups and cyanobacteria have a chlorophyll *a*-containing antenna complex functionally associated with PS I and phycobilisomes, which serve as a light-harvesting antenna in PS II [9,19]. While in cyanobacteria chlorophylls are bound directly to the A and B subunits of the PS I reaction centre, in red algae and Cryptophyta proteins related to Cab family members participate in chlorophyll *a*-binding [19]. In Prochlorophyta the light-harvesting antenna consists of both chlorophylls *a* and *b* [17] bound to proteins fundamentally different from Cab proteins. These proteins are encoded by the *IsiA* (iron stress-induced) gene [13] and are related to the CP43 protein of PS II core complex in higher plants. Interestingly, all these organisms, independently of the antenna-type, contain only one helix Hlips/Scps (Table 1 and Figure 1A). No Seps or Elips were found in these algae or cyanobacteria, suggesting that two- or three-helix Elip family members arose more recently.

Three-helix Elip proteins appeared for the first time in the green algae *Dunaliella bardawil* and *Chlamydomonas reinhardtii*, which contained one or six Elips, respectively (Table 1, Figure 1A). In the moss *Tortula ruralis* and the fern *Onoclea sensibilis*, two or one Elips were found in the nuclear genomes. A dicotyledon, *Arabidopsis thaliana*, contained two three-helix Elips, five two-helix Seps and two Ohps. The genome of

Copyright © 2002 John Wiley & Sons, Ltd. 

**Table 1.** Elip family members in various photosynthetic organisms and their predicted secondary structure. The following abbreviations are used: aa, amino acid; C, random coil; E, extended strand; H, $\alpha$−helix; ORF, open reading frame; TC, tentative consensus; TP, transit peptide. The length of chloroplast transit peptides was predicted using the TargetP program, version 1.01, and a secondary structure of proteins was analysed by the programs HNN (Hierarchical Neural Network method), DAS (Dense Alignment Surface method) and TMHMM version 2.0. All of these prediction programs are available at the web page **http://www.expasy.ch/tools/**

| Organism and protein name | Protein and transit peptide size (aa) | Number of $\alpha$−helices | Size of extra−membraneous regions | Secondary structures |
|---|---|---|---|---|
| *Prochlorococcus marinus* | | | | |
| Hlip/Scp (ORF292581−292700) | partial | 1 | n.d. | n.d. |
| Hlip/Scp (ORF320963−321103) | partial | 1 | n.d. | n.d. |
| Hlip/Scp (ORF634063−634167) | partial | 1 | n.d. | n.d. |
| Hlip/Scp (ORF702413−702544) | partial | 1 | n.d. | n.d. |
| Hlip/Scp (ORF713054−713146) | partial | 1 | n.d. | n.d. |
| Hlip/Scp (ORF777520−777597) | partial | 1 | n.d. | n.d. |
| Hlip/Scp (ORF962058−962189) | partial | 1 | n.d. | n.d. |
| Hlip/Scp (ORF1274287−1274418) | partial | 1 | n.d. | n.d. |
| Hlip/Scp (ORF1550514−1550600) | partial | 1 | n.d. | n.d. |
| Hlip/Scp (ORF1600713−1600811) | partial | 1 | n.d. | n.d. |
| *Synechococcus* sp. PCC 7942 | partial | 1 | n.d. | n.d. |
| Hlip/Scp (ORF403595−403681) | partial | 1 | n.d. | n.d. |
| Hlip/Scp (ORF489425−489565) | partial | 1 | n.d. | n.d. |
| Hlip/Scp (ORF757171−757254) | partial | 1 | n.d. | n.d. |
| Hlip/Scp (ORF830464−830550) | partial | 1 | n.d. | n.d. |
| Hlip/Scp (ORF1152233−1152361) | partial | 1 | n.d. | n.d. |
| Hlip/Scp (ORF1840310−1840429) | partial | 1 | n.d. | n.d. |
| Hlip/Scp (ORF2114479−2114619) | partial | 1 | n.d. | n.d. |
| Hlip/Scp (ORF2299709−2299873) | partial | 1 | n.d. | n.d. |
| *Nostoc* sp. PCC7120 | | | | |
| Hlip/Scp (BAB72407) | 40 | 1 | 15 | C43%, E13%, H44% |
| Hlip/Scp (BAB72472) | 72 | 1 | 48 | C51%, E18%, H31% |
| Hlip/Scp (BAB72830) | 67 | 1 | 43 | C52%, E3%, H45% |
| Hlip/Scp (BAB74053) | 56 | 1 | 32 | C46%, E9%, H45% |
| Hlip/Scp (BAB74741) | 67 | 1 | 43 | C46%, E9%, H45% |
| Hlip/Scp (BAB74742) | 67 | 1 | 43 | C48%, E6%, H46% |
| Hlip/Scp (BAB75425) | 59 | 1 | 35 | C53%, E19%, H28% |
| Hlip/Scp (BAB76961) | 59 | 1 | 35 | C42%, E24%, H34% |
| *Cyanophora paradoxa* | | | | |
| Hlip/Scp (P48367) | 49 | 1 | 25 | C31%, E12%, H57% |
| *Porphyra purpurea* | | | | |
| Hlip/Scp (AAC08241) | 48 | 1 | 24 | C35%, E23%, H42% |
| *Cyanidium caldarium* | | | | |
| Hlip/Scp (NP045110) | 43 | 1 | 19 | C30%, E0%, H70% |
| *Guillardia theta* | | | | |
| Hlip/Scp (NP050676) | 53 | 1 | 29 | C40%, E21%, H39% |
| Hlip/Scp (NP113345) | 127 | 1 | 103 | C32%, E22%, H46% |
| *Dunaliella bardawil* | | | | |
| Elip (L32871) | 172−TP40 | 3 | 63 | C46%, E7%, H47% |
| *Chlamydomonas reinhardtii* | | | | |
| Elip (TC686) | 175−TP29 | 3 | 77 | C45%, E0%, H55% |
| Elip (TC3892) | 192−TP10 | 3 | 113 | C50%, E15%, H35% |
| Elip (TC4880) | 190−TP34 | 3 | 87 | C52%, E5%, H43% |
| Elip (TC6231) | 162−TP16 | 3 | 77 | C49%, E14%, H37% |
| Elip (TC7565) | 155−TP38/partial | 3 | n.d. | n.d. |
| Elip (TC11817) | 197−TP31 | 3 | 97 | C46%, E11%, H43% |

Copyright © 2002 John Wiley & Sons, Ltd. 

**Table 1.** Continued

| Organism and protein name | Protein and transit peptide size (aa) | Number of α−helices | Size of extra−membraneous regions | Secondary structures |
|---|---|---|---|---|
| *Tortula ruralis* | | | | |
| Elip (AAK59376) | 212−TP36 | 3 | 107 | C56%, E16%, H28% |
| Elip (AAK59377) | 224−TP45 | 3 | 110 | C46%, E9%, H45% |
| *Onoclea sensibilis* | | | | |
| Elip (AAB25012) | 220−TP40/partial | 3 | 111 | C54%, E4%, H42% |
| *Oryza sativa* | | | | |
| Elip (AC007789) | 202−TP44 | 3 | 89 | C55%, E1%, H44% |
| Sep (AC122144) | 138−TP60 | 2 | 33 | C42%, E8%, H50% |
| Ohp (BAB89037) | 190−TP38 | 1 | 128 | C63%, E5%, H32% |
| Ohp (AF017356) | 157−TP41 | 1 | 92 | C67%, E2%, H31% |
| Ohp (AF017357) | 168−TP44 | 1 | 100 | C78%, E4%, H18% |
| *Hordeum vulgare* | | | | |
| Elip (CAA33726) | 167−TP35 | 3 | 63 | C46%, E2%, H52% |
| Elip (CAA33727) | 172−TP40 | 3 | 63 | C45%, E0%, H55% |
| Elip (CAA33728) | 231−TP33 | 3 | 129 | C50%, E14%, H36% |
| Elip (TC17165) | 192−TP35 | 3 | 88 | C55%, E3%, H42% |
| Sep (AL450924) | 195−TP36 | 2 | 114 | C49%, E18%, H33% |
| Sep (TC17746) | 147−TP?/partial | 2 | n.d. | n.d. |
| Sep (TC22678) | 150−TP29 | 2 | 76 | C32%, E12%, H56% |
| Sep (TC24299) | 124−TP33 | 2 | 46 | C44%, E37%, H19% |
| Ohp (TC17490) | 181−TP33 | 1 | 124 | C57%, E7%, H36% |
| Ohp (TC17908) | 108−TP38 | 1 | 46 | C56%, E7%, H37% |
| *Arabidopsis thaliana* | | | | |
| Elip (NP188923) | 195−TP44 | 3 | 82 | C52%, E11%, H37% |
| Elip (NP567438) | 193−TP41 | 3 | 83 | C50%, E5%, H45% |
| Sep (NP199522) | 254−TP38 | 2 | 171 | C49%, E11%, H40% |
| Sep (NP565524) | 202−TP21 | 2 | 136 | C42%, E16%, H42% |
| Sep (NP567532) | 262−TP39 | 2 | 178 | C45%, E10%, H45% |
| Sep (NP567794) | 157−TP52 | 2 | 60 | C33%, E20%, H47% |
| Sep (NP567958) | 146−TP43 | 2 | 58 | C47%, E18%, H35% |
| Ohp (NP195832) | 110−TP40 | 1 | 46 | C44%, E7%, H49% |
| Ohp (NP564432) | 172−TP42 | 1 | 106 | C57%, E8%, H35% |
| *Solanum tuberosum* | | | | |
| Elip (TC30191) | 194−TP40 | 3 | 85 | C54%, E6%, H40% |
| Sep (TC29806) | 264−TP56 | 2 | 163 | C51%, E5%, H44% |
| Sep (TC39042) | 141−TP61 | 2 | 35 | C36%, E14%, H50% |
| Sep (TC39823) | 192−TP31 | 2 | 116 | C39%, E23%, H38% |
| Ohp (TC37193) | 169−TP66 | 1 | 79 | C30%, E9%, H61% |
| Ohp (TC39532) | 168−TP79 | 1 | 65 | C35%, E9%, H56% |

a monocotyledon, *Oryza sativa*, contained one Elip, one Sep and three different Ohps (Table 1, Figure 1). We also searched the EST database of *Solanum tuberosum* and *Hordeum vulgare* for the presence of Elip family members. On the basis of the deduced amino acid sequences and topological predictions one Elip, three Seps and two Ohps were found in *S. tuberosum*, while *H. vulgare* contained four Elips, four Seps and two Ohps (Table 1, Figure 1). All Elip family members in green algae, mosses, ferns and higher plants investigated so far are nuclear-encoded proteins posttranslationally imported into chloroplasts [1]. This indicates that during evolution *Elip* genes have undergone translocation from plastids to the nucleus and have further evolved into topologically different proteins. It is generally accepted that chloroplasts are derived from a single cyanobacterial ancestor. Of

Copyright © 2002 John Wiley & Sons, Ltd.

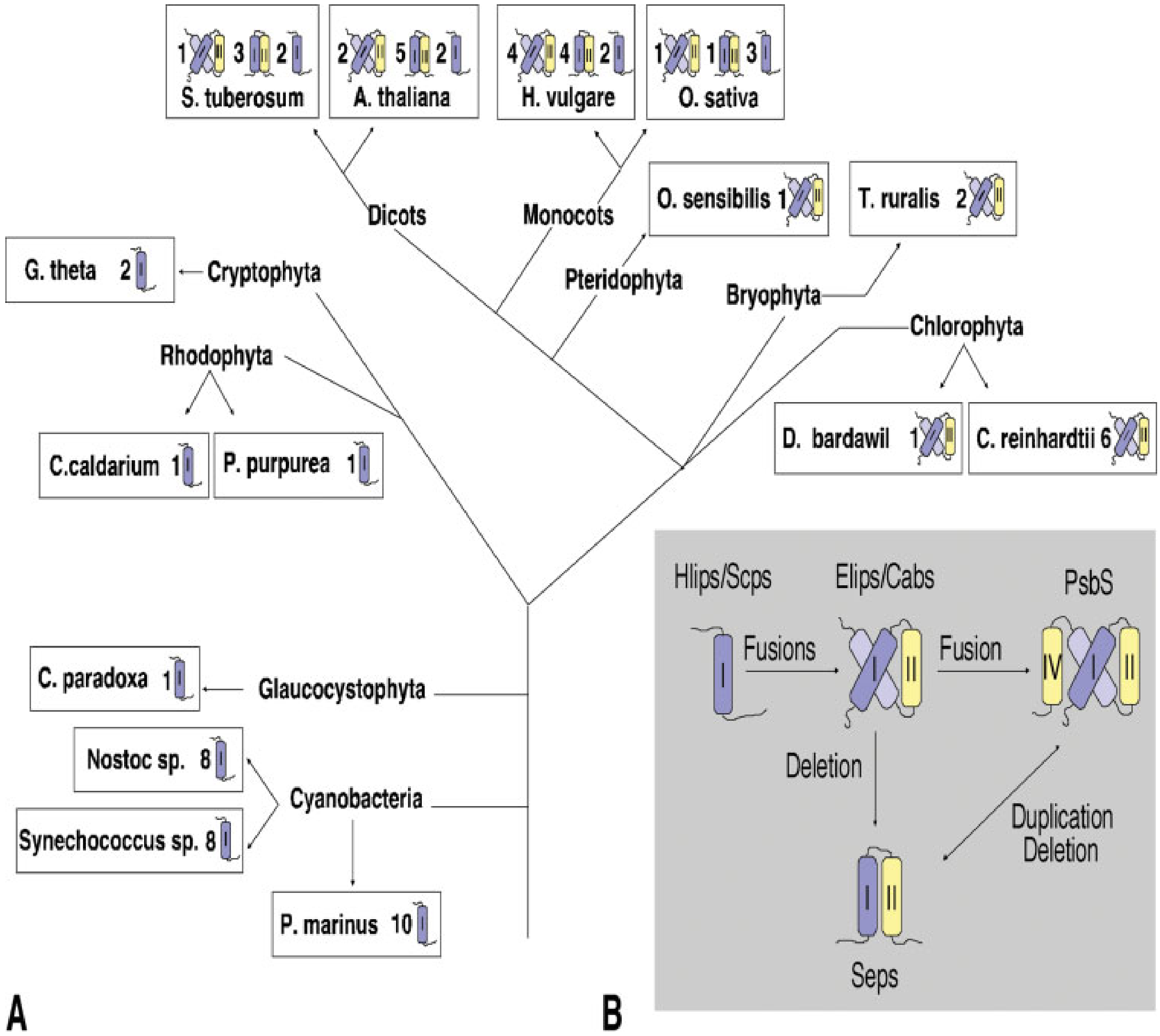

**Figure 1.** (A) Distribution of Elip family members in oxygenic photosynthetic organisms. Phylogenetic tree of major pro- and eukaryotic groups, drawn according to [18], where the presence of Elip family members was analysed. Numbers of Elip family members and the predicted topological structure are shown schematically for each organism analysed. (B) Model for the hypothetical evolution of Elips and Cab proteins. The members of these families are represented schematically by their predicted topological structure and arrows indicate the direction of evolution. The conserved transmembrane helices are marked in blue and the polymorphic helix in yellow. Sequences used in the data set are from the following databases: *Prochlorococcus marinus*, *Synechococcus* sp. PCC7942, *Nostoc* sp. PCC7120, *Cyanophora paradoxa*, *Porphyra purpurea*, *Cyanidium caldarium*, *Guillardia theta*, *Dunaliella bardawil*, *Chlamydomonas reinhardtii*, *Tortula ruralis*, *Onoclea sensibilis*, *Oryza sativa*, *Hordeum vulgare*, *Arabidopsis thaliana* and *Solanum tuberosum*, as shown in Table 1

the 3000 genes present in the cyanobacterium *Synechocystis* PCC6803, only 100–200 are found on plastid genomes, indicating that a massive transfer of genes to the nucleus occurred following endosymbiosis and establishment of the plastid [3].

The antenna system in green algae and higher plants is composed of Cab proteins associated with PS I and PS II that are encoded by multigene families consisting of 21 different members, as was reported for *A. thaliana* [11]. The Cab family in higher plants also includes the PsbS protein with four transmembrane helices, which is located in PS II [5]. Searches in the databases revealed that one *PsbS* gene has been annotated in the *A. thaliana*

Copyright © 2002 John Wiley & Sons, Ltd. 

(GenBank Accession No. NP175092), *S. tuberosum* (EST TC35092) and *H. vulgare* (EST TC14735) genomes. Interestingly, *O. sativa* contained two different *PsbS* genes (GenBank Accession Nos BAA12337 and BAB64099).

### Hypothetical evolution of Elips and Cab proteins

According to a model proposed for the structural evolution of antenna systems [8] two Hlip/Scp-type genes fused during evolution, resulting in the generation of a two-helix ancestor of Cab proteins. Recently, two-helix Seps were described from *A. thaliana* [10] and proposed to be a missing link between one-helix Hlips of prokaryota and three-helix Elips and Cab proteins of higher plants and green algae. An internal gene duplication of a two-helix Sep-like protein was thought to result in the creation of a four-helix intermediate, the PsbS protein. This theory is supported by the fact that transmembrane helices I and III, and II and IV of the PsbS protein are clearly related [5]. Finally, the deletion of the fourth helix of the PsbS protein has given rise to the three-helix ancestor of Elip and Cab proteins [8]. According to this scenario, the ancient antenna systems seemed to be composed of light stress proteins, the function of which was not light-harvesting but the dissipation of absorbed energy in the form of heat or fluorescence [16]. In our study, there is no evidence for the existence of two-helix Seps or four-helix PsbS in green algae, mosses or ferns, although they contain three-helix Elips and Cab-type antenna systems (Figure 1A). Cyanobacteria and more primitive algae, such as Rhodophyta, Cryptophyta or Glaucocystophyta, contain only one-helix Hlips/Scps.

On the basis of our data we propose an alternative model of Elip and Cab protein evolution in eukaryota (Figure 1B). Three-helix Elips and Cab proteins could arise in parallel by a double fusion of the ancestral Hlip/Scp-type of proteins with an unknown protein, from which a second polymorphic helix originated. The consecutive deletion of the third conserved transmembrane helix of Elips would result in the formation of two-helix Seps, which in turn could duplicate and fuse to form the four-helix PsbS protein. Alternatively, an additional fusion with an unknown protein could form the PsbS protein. However, conservation of amino acid composition in helices I and III, and II and IV of the PsbS protein speaks in favour of the first scenario.

### Acknowledgements

We would like to thank Lars Hiertas Minne, the European Science Foundation, and Liljevalch Stipendiefond for financial support given to MH. This work was supported by research grants from the Swedish Research Council (B5107-20005512, B5101-20005540, S614946/2000 and S629-2002-1131) to I.A.

### References

1. Adamska I. 2001. The Elip family of stress proteins in the thylakoid membrane of pro- and eukaryota. In *Advances in Photosynthesis and Respiration — Regulation of Photosynthesis*, vol 11, Aro E-M, Andersson B (eds). Kluwer Academic: Dordrecht, Boston, London; 487–505.
2. Adamska I, Roobol-Bóza M, Lindahl M, Andersson B. 1999. Isolation of pigment-binding early light-inducible proteins from pea. *Eur J Biochem* **260**: 453–460.
3. Douglas SE. 1998. Plastid evolution:origins, diversity, trends. *Curr Opin Genet Dev* **8**: 655–661.
4. Durnford DG, Deane JA, Tan S, McFadden GI, Gantt E, Green BR. 1999. A phylogenetic assessment of the eukaryotic light-harvesting antenna proteins with implications for plastid evolution. *J Mol Evol* **48**: 59–68.
5. Funk C. 2001. The PsbS protein: A Cab protein with a function of its own. In *Advances in Photosynthesis and Respiration — Regulation of Photosynthesis*, vol 11, Aro E-M, Andersson B (eds). Kluwer Academic: Dordrecht, Boston, London; 453–467.
6. Funk C, Vermaas W. 1999. A cyanobacterial gene family coding for single-helix proteins resembling part of the light-harvesting proteins from higher plants. *Biochemistry* **38**: 9397–9404.
7. Green BR, Kühlbrandt W. 1995. Sequence conservation of light-harvesting and stress-response proteins in relation to the three-dimensional molecular structure of LHCII. *Photosynth Res* **44**: 139–148.
8. Green BR, Pichersky E. 1994. Hypothesis for the evolution of three-helix chlorophyll *a/b* and chlorophyll *a/c* light-harvesting antenna proteins from two-helix and four-helix ancestors. *Photosynth Res* **39**: 149–162.
9. Grossman AR, Schäfer MR, Chiang GG, Collier JL. 1993. The phycobilisome, a light-harvesting complex responsive to environmental conditions. *Microbiol Rev* **57**: 725–749.
10. Heddad M, Adamska I. 2000. Light stress-regulated two-helix proteins in *Arabidopsis thaliana* related to the chlorophyll *a/b*-binding gene family. *Proc Natl Acad Sci USA* **97**: 741–746.
11. Jansson S. 1999. A guide to the identification of the *Lhc* genes and their relatives in *Arabidopsis*. *Trends Plant Sci* **4**: 236–240.
12. Kühlbrandt W, Wang DN, Fujiyoshi Y. 1994. Atomic model of plant light-harvesting complex by electron crystallography. *Nature* **367**: 614–621.

Copyright © 2002 John Wiley & Sons, Ltd. 

13. LaRoche J, van der Staay GWM, Partensky F, *et al*. 1996. Independent evolution of the prochlorophyte and green plant chlorophyll *a/b* light-harvesting proteins. *Proc Natl Acad Sci USA* **93**: 15244–15248.
14. Löffelhardt W, Bohnert H. 1994. Molecular biology of cyanelle. In *The Molecular Biology of Cyanobacteria*, Bryant DA (ed.). Kluwer Academic: Dordrecht, Boston, London; 65–89.
15. Miroshnichenko-Doglanov NA, Bhaya D, Grossman A. 1994. Cyanobacterial protein with similarity to the chlorophyll *a/b* binding protein of higher plants: Evolution and regulation. *Proc Natl Acad Sci USA* **92**: 636–640.
16. Montané MH, Kloppstech K. 2000. The family of light-harvesting-related proteins (LHCs, ELIPs, HLIPs): was the harvesting of light their primary function? *Gene* **258**: 1–8.
17. Post AF, Bullerjahn GS. 1994. The photosynthetic machinery in Prochlorophytes: structural properties and ecological significance. *FEMS Microbiol Rev* **13**: 393–413.
18. Tomitani A, Okada K, Miyashita H, Matthijs HC, Ohno T, Tanaka A. 1999. Chlorophyll *b* and phycobilins in the common ancestor of cyanobacteria and chloroplasts. *Nature* **400**: 159–162.
19. Wolfe GR, Cunningham FX, Durnford D, Green BR, Gantt E. 1994. Evidence for a common origin of chloroplasts with light-harvesting complexes of different pigmentation. *Nature* **367**: 566–568.

Copyright © 2002 John Wiley & Sons, Ltd. 
